# Artificial Intelligence for the Diagnosis and Management of Neurodegenerative Diseases: A Comprehensive Review With an Emphasis on Parkinson’s and Alzheimer’s Diseases

**DOI:** 10.7759/cureus.112325

**Published:** 2026-07-09

**Authors:** SriLakshmi Pravallika Pinnelli, Surendra Babu T, Jayashankar CA, Venkata BharatKumar Pinnelli, Ganaraja V Harikrishna, Vamsi Krishna Mudamanchu, Koshy T Sam, Divyadharshini MS, Praful Bhupathiraju, Atul S Sucharitha, Venkataramana Kandi

**Affiliations:** 1 Computer Sciences and Engineering, New Horizon College of Engineering, Bengaluru, IND; 2 Anatomy, Vydehi Institute of Medical Sciences and Research Centre, Bengaluru, IND; 3 Internal Medicine, Vydehi Institute of Medical Sciences and Research Centre, Bengaluru, IND; 4 Biochemistry, Vydehi Institute of Medical Sciences and Research Centre, Bengaluru, IND; 5 Neurology, National Institute of Mental Health and Neurosciences, Bengaluru, IND; 6 Neurology, RVM Institute of Medical Sciences and Research Centre, Siddipet, IND; 7 Neurology, Vydehi Institute of Medical Sciences and Research Centre, Bengaluru, IND; 8 General Medicine, Vydehi Institute of Medical Sciences and Research Centre, Bengaluru, IND; 9 Clinical Microbiology, Prathima Institute of Medical Sciences, Karimnagar, IND

**Keywords:** alzheimer's disease (ad), artificial intelligence, biomarkers, deep learning, digital phenotyping, ethics, machine learning, neurodegenerative diseases, neuroimaging, parkinson' s disease

## Abstract

Artificial intelligence (AI) is rapidly transforming research in neurodegenerative diseases, yet its clinical translation remains limited. We conducted a structured literature search across Google Scholar, PubMed, Scopus, and Web of Science, screening studies published between 2015 and April 2026 that applied machine learning (ML), deep learning (DL), and multimodal data integration to neuroimaging, biomarkers, and digital phenotyping. Our analysis revealed that AI models demonstrate strong potential for differentiating disease subtypes, predicting progression, and enhancing diagnostic accuracy, with notable advances in neuroimaging interpretation, fluid biomarker analysis, and wearable sensor data. In Parkinson’s disease (PD), digital phenotyping through gait, speech, and handwriting analysis has enabled sensitive monitoring, while in Alzheimer’s disease (AD), AI applied to imaging and plasma biomarkers has improved risk stratification. Despite these advances, barriers such as dataset heterogeneity, label noise, lack of external validation, and ethical concerns regarding bias, transparency, and patient trust persist. We conclude that while AI holds promise to revolutionize the care of PD and AD, real-world adoption requires multicenter validation, standardized reporting frameworks, regulatory guidance, and interdisciplinary collaboration, alongside prospective trials that embed AI tools into clinical workflows to ensure safety, equity, and effectiveness.

## Introduction and background

Neurodegenerative disorders are among the leading causes of chronic disability and dependency worldwide, with their impact intensifying as populations age. The urgency of translating artificial intelligence (AI) into clinical practice in this domain arises not only from rapid algorithmic progress but also from the pressing public health need to address diagnostic delays and the escalating socioeconomic burdens. Parkinson’s disease (PD) and Alzheimer’s disease (AD) together account for the majority of progressive motor and cognitive impairment, caregiver strain, and long-term institutional care needs [1-3]. These conditions impose substantial societal costs through lost productivity, downstream medical expenses, and reliance on informal caregiving. Moreover, inequities in access to specialist assessment and advanced diagnostics further exacerbate late presentation and delayed intervention [2,4].

A central challenge in neurodegenerative medicine is the long latency between the onset of pathophysiological processes and the emergence of overt symptoms. In PD, this may manifest as nonspecific prodromal features such as hyposmia, constipation, depression, or rapid eye movement (REM) sleep behavior disorder years before motor signs [5,6]. In AD, it may present as mild cognitive impairment (MCI), a heterogeneous transitional state marked by subtle decline and biomarker abnormalities across extended preclinical phases [5,7]. This diagnostic delay limits opportunities for early disease-modifying therapies and timely trial enrollment. At the same time, traditional approaches relying on clinical criteria remain vulnerable to inter-rater variability, comorbidities, and atypical or mixed presentations that complicate differentiation because of overlapping nonspecific motor, autonomic, and neuropsychiatric features, as well as atypical parkinsonian disorders and secondary cognitive syndromes such as multiple system atrophy (MSA) and related conditions [6-8].

Neuroimaging and fluid biomarkers have improved diagnostic confidence. MRI reveals regional patterns of atrophy, positron emission tomography (PET) quantifies amyloid and tau burden, and cerebrospinal fluid (CSF) or plasma assays provide biological specificity. However, their widespread adoption is limited by high cost, invasiveness, assay variability, and reliance on expert interpretation [5,7,9,10]. In PD, the absence of a single confirmatory biomarker necessitates reliance on disease progression, treatment response, and supportive imaging, which remain ambiguous in the early stages [3,6]. Consequently, research has shifted toward automated differentiation systems and digital biomarkers, including gait, speech, and handwriting signals, to capture early dysfunction and improve diagnostic accuracy [11].

AI and machine learning (ML) approaches offer a promising solution by uncovering predictive patterns within complex, high-dimensional data [1,12]. These methods range from traditional supervised learning (e.g., logistic regression, support vector machines, random forests, and gradient boosting) to deep learning (DL) architectures such as convolutional neural networks and transformers, which can learn directly from imaging, audio, or time-series sensor data [9,12]. Their potential lies in detecting subtle multimodal markers, integrating diverse data sources such as genetics, biomarkers, digital phenotyping, and clinical features, enabling personalized risk assessment and staging, and supporting continuous monitoring through wearables such as smartwatches and smartphones [10,13]. In AD, these multimodal architectures are increasingly leveraged to predict clinical trajectories and the progression from mild cognitive impairment to overt dementia [14]. Applied appropriately, AI can reduce diagnostic latency, strengthen specialist decision-making, and promote individualized care strategies in both PD and AD.

Nevertheless, caution is warranted when interpreting reported performance metrics. Many studies rely on curated datasets with limited demographic diversity, uniform protocols, and case-control designs that inflate apparent accuracy compared with real-world populations [8-14]. Methodological pitfalls include data leakage, inadequate handling of scanner and site variability, class imbalance, and an overemphasis on discrimination metrics while neglecting calibration, uncertainty quantification, and clinical utility [15]. Additional challenges arise from age-related confounding, cerebrovascular comorbidity, medication effects, and socioeconomic disparities that influence both access and outcomes [4,8]. Ethical concerns remain central: algorithmic bias may exacerbate inequities, opaque "black box" models risk undermining clinical trust, and privacy issues intensify with large-scale multimodal data collection [16].

**Figure 1:**
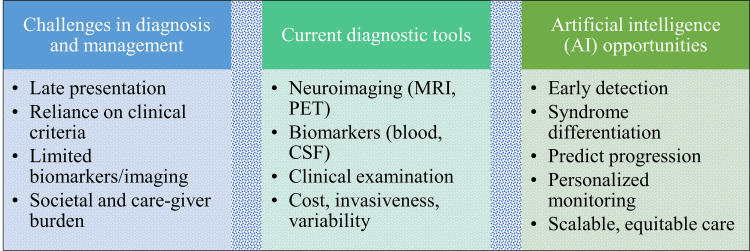
Artificial intelligence in neurodegenerative diseases: from diagnostic challenges to emerging opportunities AI: artificial intelligence; MRI: magnetic resonance imaging; PET: positron emission tomography; CSF: cerebrospinal fluid Image credit: Venkataramana Kandi. This image is an original author-created schematic using Microsoft Word/PowerPoint (Microsoft® Corp., Redmond, WA) and was not generated using AI

This review therefore synthesizes current evidence on AI and ML applications in PD and AD, focusing on four domains: (i) early detection and differential diagnosis, (ii) prediction of disease progression and prognosis, (iii) risk stratification and identification of high-risk patients, and (iv) individualized care and monitoring strategies enabled by multimodal data [1,13,14]. By critically appraising methodological quality, generalizability, interpretability, and ethical considerations, we aim to provide a balanced framework for evaluating current capabilities and identifying future research priorities in clinical neurology (Figure 1).

## Review

Methodology

This structured narrative review examined applications of AI and ML in PD and AD, with a particular emphasis on clinical translation. To ensure the highest standards of methodological transparency, reproducibility, and clinical safety, the critical appraisal of the literature synthesized in this review was systematically aligned with established international consensus guidelines for artificial intelligence in healthcare. Specifically, clinical trial evaluation targets and workflow integration paradigms were benchmarked against the Consolidated Standards of Reporting Trials for Artificial Intelligence (CONSORT-AI) extension. Concurrently, multivariable prediction models, prognostic algorithms, and ML-driven risk stratification frameworks, such as those evaluating the timeline of MCI-to-AD conversion, were critically analyzed using the Transparent Reporting of a Multivariable Prediction Model for Individual Prognosis or Diagnosis for Artificial Intelligence (TRIPOD-AI) statement.

Comprehensive compliance mapping tables demonstrating the integration of these dual frameworks within our systematic synthesis are provided [13] (see Appendices). In parallel with these methodological frameworks, recently published consensus statements and regulatory guidance have underscored the requirements for the safe and effective clinical deployment of AI-based decision support systems. The CONSORT-AI and TRIPOD-AI extensions emphasize transparent reporting and external validation, while regulatory agencies such as the U.S. Food and Drug Administration (FDA) and the European Medicines Agency (EMA) have issued guidance on software as a medical device (SaMD), highlighting explainability, bias mitigation, and post-market surveillance [13,14]. These evolving standards provide essential context for interpreting the translational potential of AI in neurodegenerative disease care.

We searched PubMed/MEDLINE, Scopus, Web of Science Core Collection, and Google Scholar, supplemented by manual reference screening. Search terms combined controlled vocabulary and free-text keywords for PD, AD, and dementia subtypes, alongside AI/ML concepts including ML, DL, convolutional neural networks, and transformers, as well as modality-specific terms such as MRI, PET, CSF, wearables, speech, and gait. Literature published from January 2015 to April 2026 was reviewed, with landmark studies published before 2015 included for contextual reference. Eligible studies included human investigations applying AI or ML to PD or AD for diagnosis, prognosis, risk prediction, monitoring, or management and reporting quantitative performance metrics. Exclusion criteria included animal studies, non-neurological indications, theoretical or commentary articles, insufficient methodological detail, and duplicate publications. Screening was performed at the title and abstract level, followed by full-text review, with disagreements resolved conservatively. Priority was given to studies that incorporated external validation, clinically realistic comparator groups, transparent reporting of preprocessing and model tuning, and clinically interpretable metrics.

Data extraction captured study design, participant characteristics, modalities, preprocessing steps, model architecture, training and validation strategies, performance metrics, interpretability, calibration, subgroup analyses, and intended clinical applications. Critical appraisal focused on risks of bias, including data leakage, confounding, and spectrum bias, as well as generalizability, calibration, subgroup performance, uncertainty estimation, and ethical considerations. Findings were synthesized narratively by disease (PD vs. AD) and clinical task, including diagnosis, prognosis, risk stratification, and monitoring. Meta-analysis was not performed due to heterogeneity. Limitations include the inherent constraints of narrative reviews, potential publication bias, selective reporting, and reliance on preprints that require careful interpretation.

**Figure 2:**
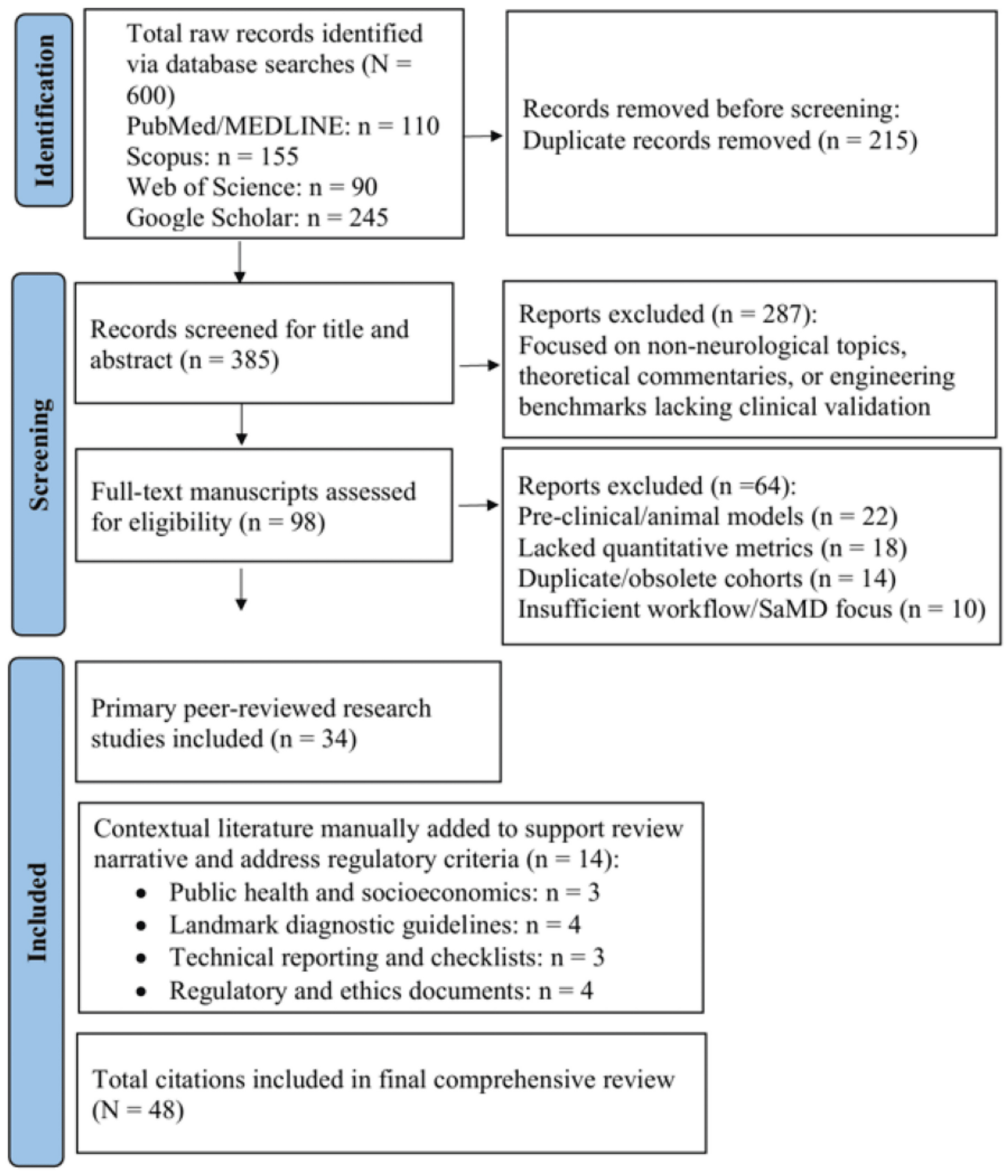
Flowchart of the literature selection process AI: artificial intelligence; SaMD: software‑as‑a‑medical‑device

The comprehensive selection, filtering, and distribution of literature utilized in this study are illustrated in Figure 2. A multi-stage screening pipeline tracking initial records (N = 600) from January 2015 to April 2026 was employed to identify the 34 primary clinical AI research studies evaluated herein. This core dataset was supplemented by 14 contextual, diagnostic, and regulatory reference works, yielding a final bibliographic set of 48 citations (Figure 2).

While individual applications of AI in PD and AD demonstrate promising technical performance, the true value of these approaches lies in their clinical interpretation and translational potential. A critical synthesis of the literature reveals several recurring themes. First, AI systems consistently outperform traditional diagnostic heuristics in controlled datasets; however, their utility in real-world practice depends on external validation, calibration, and integration into clinician workflows [13,14]. Second, multimodal approaches that combine imaging, fluid biomarkers, and digital phenotyping appear to be the most clinically relevant, as they address the heterogeneity of neurodegenerative diseases and provide a more holistic framework for risk stratification [9,10,16]. Third, the clinical impact of AI should be judged not only by accuracy metrics but also by its ability to reduce diagnostic latency, guide individualized management, and improve patient-centered outcomes, such as caregiver support and treatment optimization [2,4,13].

Finally, ethical and regulatory considerations, including bias mitigation, transparency, and patient trust, are not peripheral issues but rather central determinants of whether AI tools will be adopted safely and equitably in neurology practice [13]. Taken together, these insights underscore that AI applications in neurodegenerative diseases must be evaluated less as isolated technical achievements and more as components of evolving clinical ecosystems. The emphasis should therefore shift from reporting performance metrics alone to interpreting how these tools reshape diagnostic pathways, prognostic counseling, and long-term care strategies (Table 1).

**Table 1:** AI promise vs. clinical reality in neurodegenerative diseases AI: artificial intelligence; SaMD: software as a medical device

AI promise	Clinical reality/interpretation
High diagnostic accuracy in controlled datasets	Performance often declines in real-world settings due to heterogeneity, comorbidities, and site variability; external validation is essential [13,14]
Early detection through digital biomarkers (gait, speech, handwriting)	Useful for screening and monitoring, but confounded by age, comorbidities, and device variability; requires careful calibration for clinical adoption [2,12]
Multimodal integration of imaging, fluid biomarkers, and genetics	Offers holistic risk stratification and individualized staging, but implementation is limited by cost, accessibility, and standardization challenges [9,10,16]
Deep learning models uncovering subtle imaging patterns	Can detect high-dimensional features beyond human interpretation, yet lack transparency and interpretability may undermine clinician trust [13]
Continuous monitoring via wearables and smartphones	Enables patient-centered care outside clinics, but raises concerns about adherence, digital literacy, and privacy protections [2,14]
Rapid translation into clinical workflows	Regulatory, ethical, and workflow integration barriers remain; adoption depends on alignment with consensus frameworks and SaMD guidance [13,14]

Artificial intelligence in Parkinson’s disease

AI applications in PD include (i) support for early and differential diagnosis, (ii) objective quantification of motor and non-motor features, (iii) prediction of near-term progression and clinically meaningful outcomes, and (iv) longitudinal monitoring to inform individualized management [1,13]. The clinical need is significant since PD is variable in its presentation, development, and response to medication, and early-stage clinical symptoms may overlap with essential tremor and atypical parkinsonian disorders [5,7]. As a result, the most clinically relevant PD-oriented AI research has tended to focus on tasks that are difficult to standardize in routine practice, such as recognizing subtle patterns in neuroimaging, quantifying motor behavior outside the clinic, and combining multiple weak signals into a more reliable diagnostic or prognostic estimate [14,17].

Neuroimaging-Based AI Models

Neuroimaging has long been central to the evaluation of Parkinson’s disease, but its clinical utility is often constrained by the subtlety of findings and variability across sites. AI approaches offer a means of extracting clinically meaningful patterns from both structural and functional imaging; however, their value lies less in raw accuracy and more in how they address diagnostic uncertainty. For example, diffusion-weighted MRI (DWI) and diffusion tensor imaging (DTI) can reveal microstructural changes that are not visible on conventional sequences, while dopaminergic imaging, such as dopamine transporter single-photon emission computed tomography (DaT-SPECT), provides functional evidence of presynaptic deficiency [11,15,18]. However, the challenge is not simply the detection of abnormalities; it is whether these tools can reliably differentiate PD from atypical parkinsonian syndromes in real-world practice, where comorbidities and mixed phenotypes are common [7,8].

AI applications in neuroimaging generally fall into two paradigms: feature-based pipelines and end-to-end deep learning. While feature-based models leverage predefined metrics (e.g., diffusion indices and uptake ratios) and can achieve strong performance in curated datasets, they risk oversimplifying complex disease biology. Deep learning, by contrast, can uncover high-dimensional representations directly from raw images but requires large, harmonized datasets and rigorous external validation to avoid inflated results [14,17]. The critical issue is not which architecture performs better in controlled experiments, but rather which approach produces outputs that clinicians can interpret and act upon.

From a translational perspective, the most informative studies are those that evaluate algorithms against clinically realistic comparison groups, including patients with early disease, overlapping syndromes, or vascular comorbidities, rather than clean case-control designs [14,19]. In these contexts, probabilistic estimates, uncertainty quantification, and region-level contributions are more valuable than binary labels because they support nuanced decision-making. Ultimately, neuroimaging-based AI in PD should be viewed as an assistive layer that standardizes interpretation and reduces diagnostic delay, rather than as a stand-alone diagnostic replacement. Its success will depend on harmonization across scanners, transparency of outputs, and demonstration of its added value to patient-centered outcomes.

Automated Imaging Differentiation in Parkinsonism (AID-P) and Diffusion Weighted MRI

Automated Imaging Differentiation in Parkinsonism (AID-P) illustrates both the promise and challenges of imaging-based AI in clinical neurology. Reported diagnostic accuracies approaching 96% in multicenter evaluations highlight its potential to standardize interpretation across sites and reduce diagnostic delay [15]. However, the true clinical value of AID-P lies not in its headline accuracy figures, but in its ability to support clinicians when diagnostic uncertainty is greatest, such as in distinguishing idiopathic PD from atypical parkinsonian syndromes, where prognosis and therapeutic response diverge significantly. By providing consistent and reproducible outputs, AID-P can facilitate earlier counseling and therapy decisions, but only if its performance translates reliably across diverse patient populations.

Implementation considerations underscore why caution is essential. Diffusion-derived features are highly sensitive to acquisition parameters, scanner hardware, and preprocessing choices, meaning that harmonization across sites is a prerequisite for generalizability [14]. Moreover, the reference standard for diagnosis is often clinical rather than neuropathological, introducing label noise that constrains achievable performance and complicates interpretation when algorithmic predictions diverge from subsequent clinical diagnoses [7]. For clinicians, interpretability is critical: probabilistic estimates, uncertainty quantification, and region-level contributions are far more useful than binary outputs because imaging represents only one component of the diagnostic work-up [20]. Finally, the intended use case must be clearly defined. A triage tool designed to flag atypical parkinsonism for referral may tolerate different trade-offs in sensitivity and specificity than a system intended to guide treatment planning or determine trial eligibility [13].

Gait, Speech, Handwriting, and Wearable Sensor Analysis

Digital biomarkers have emerged as one of the most clinically promising avenues for AI in Parkinson’s disease, precisely because they capture motor and speech features that are difficult to standardize in routine practice. Wearable inertial measurement units (IMUs) and smartphone-based tasks can quantify tremor, bradykinesia, gait variability, and freezing episodes with far greater granularity than intermittent clinic visits [12,21-23]. Their true value lies in enabling continuous, home-based monitoring that reflects daily fluctuations and medication “on/off” states, thereby providing clinicians with a richer understanding of disease dynamics than traditional assessments.

From a translational perspective, these tools are most impactful when used as scalable screening and monitoring mechanisms rather than stand-alone diagnostics. Voice and speech analysis, for example, can detect hypophonia and dysarthria years before overt motor symptoms, but their clinical utility is realized through longitudinal tracking of subtle changes rather than binary classification [24]. Similarly, handwriting and drawing tasks capture micrographia and fine motor decline, yet their greatest contribution lies in serving as low-threshold, community-level assessments that can trigger timely specialist referral [25].

The integration of multimodal behavioral streams with fluid biomarkers and genetic risk profiles further strengthens predictive models, but significant challenges remain. Selection bias due to technology access, variability in adherence, and device heterogeneity can degrade performance outside controlled settings [2,12]. Moreover, contextual factors, such as age, arthritis, neuropathy, or microphone quality, must be accounted for to avoid the misinterpretation of signals [2,14]. Ethical and regulatory concerns are equally central: passive monitoring generates large volumes of sensitive behavioral data, underscoring the need for robust consent frameworks, privacy protections, and clear governance structures [2].

Multimodal AI Frameworks

Multimodal AI frameworks represent an important step toward clinical robustness by integrating complementary signals, such as video-based motor assessments, voice analysis, and gait features from wearable sensors [26]. Reported accuracies of up to 94% in early detection are encouraging; however, these figures are highly dependent on dataset design, the prevalence of confounders, and whether evaluation is performed on independent cohorts [14,26]. The true clinical value of multimodal models lies not in headline performance metrics, but in their ability to enhance diagnostic confidence in borderline cases, provide stable longitudinal measures linked to meaningful outcomes, and integrate seamlessly into existing workflows [13].

From a translational perspective, tiered deployment strategies are particularly compelling. Low-cost digital screening in community or primary care settings can identify individuals at higher risk, who may then undergo specialist review or targeted imaging. Such triage workflows can help mitigate the shortage of movement disorder specialists by filtering out clear negatives and reserving expert resources for complex or ambiguous presentations. Importantly, the clinical utility of multimodal AI depends on the explicit definition of its intended role, whether as a screening tool, monitoring platform, or decision support system for treatment planning. Each use case requires different trade-offs in sensitivity, specificity, and interpretability.

Diagnostic Accuracy and Clinical Applicability

Reported diagnostic accuracy in AI studies of PD is often summarized as a single performance score, but such figures can be misleading if interpreted without clinical context. Applicability depends not only on algorithm design but also on disease prevalence in the target population and the consequences of false-positive and false-negative results [27,28]. For screening tools, calibration, decision thresholds, and downstream implications are as important as discrimination metrics, including considerations of whether earlier referral meaningfully alters management or patient outcomes [13,21].

Successful clinical adoption will require more than retrospective accuracy reports. Prospective evaluation, demonstration of value for patient-centered outcomes, and seamless integration into clinician workflows are essential. Rather than replacing clinical judgment, AI is most likely to succeed as an assistive layer by standardizing quantitative measures such as gait variability or tremor severity and supporting early identification of high-risk phenotypes. In this role, clinicians remain responsible for synthesizing diagnostic data and managing uncertainty, while AI provides reproducible, scalable inputs that enhance decision-making [13] (Table 2).

**Table 2:** Common PD data modalities used in AI systems and typical clinical tasks PD: Parkinson’s disease; MRI: magnetic resonance imaging; DWI: diffusion-weighted imaging; DTI: diffusion tensor imaging; ROI: region of interest; DaT-SPECT: dopamine transporter single photon emission computed tomography; PET: positron emission tomography; IMU: inertial measurement unit; ENT: ear, nose, and throat

Modality	AI Inputs	Representative clinical tasks and considerations
MRI (structural, DWI/DTI)	Voxel/ROI features; diffusion indices; learned image representations	Differential diagnosis (PD vs. atypical parkinsonism); requires harmonization across scanners and careful reference standards [11,15]
Nuclear imaging (DaT-SPECT/PET)	Uptake ratios; spatial uptake patterns	Support for dopaminergic deficit vs. non-degenerative tremor; limited by access/cost and protocol variability [18]
Wearables/IMUs	Time-series acceleration/gyroscope features	Tremor quantification, gait/fall risk, freezing of gait detection; sensitive to adherence and device placement [12,22]
Smartphone tasks	Tapping, gait, reaction-time tasks	Remote screening and monitoring at scale; selection bias and device heterogeneity are common challenges [23]
Speech/voice	Acoustic features; embeddings from spectrograms	Early detection and severity tracking; confounded by language, microphone quality, and comorbid ENT disease [24]
Handwriting/drawing	Kinematics; image features	Micrographia and fine motor control assessment; influenced by education, arthritis, and task instructions [25]

Artificial intelligence in Alzheimer’s disease

AI applications in AD are shaped by two key realities: dementia represents the late-stage clinical outcome of a long biological process, and an expanding set of biomarkers, including imaging, fluid, and genetic measures, can capture this biology but remain unevenly accessible and complex to interpret in clinical practice [10,16]. Within this context, AI plays clinically meaningful roles by detecting subtle disease signatures in neuroimaging for earlier diagnosis and differentiation, integrating biomarkers across domains to strengthen diagnostic confidence and staging, predicting progression, such as conversion from MCI to dementia and time-to-milestone outcomes, and enabling risk stratification strategies that support personalized monitoring and prevention pathways [13,20]. A key nuance in evaluating AI performance in AD is that the definition of “ground truth” depends on the chosen reference standard. Some studies train models using clinical labels, while others rely on biomarker-confirmed definitions, such as the amyloid, tau, neurodegeneration (AT(N)) framework, which alters both class composition and apparent accuracy [10]. Moreover, because mixed pathology is common in older adults, even well-calibrated models may yield uncertain outputs when applied to real-world cohorts that differ from the curated research datasets used for development [8].

MRI-Based Deep Learning Models

Structural MRI remains one of the most accessible modalities for evaluating AD and is routinely used to exclude alternative causes while providing supportive evidence through characteristic medial temporal and association cortex atrophy [16]. AI, particularly DL, has been applied extensively to MRI because convolutional architectures can capture distributed atrophy patterns that may be overlooked by region-based summary measures [11]. Reported accuracies for binary classification tasks, such as distinguishing AD from cognitively normal controls, are often high in curated datasets, with EfficientNet and related convolutional networks demonstrating strong performance [9]. However, these findings must be interpreted cautiously. Heavy reliance on standardized research datasets, such as ADNI, risks inflating performance due to cohort-specific scanner and site effects [14,17,29]. From a clinical standpoint, this dependency represents a transportability barrier: models calibrated on optimized research inputs may fail in community memory clinics where comorbidities, diverse demographics, and older hardware are more prevalent.

The critical issue is therefore not whether a model can achieve high accuracy under ideal conditions, but whether it can maintain stability under distribution shifts and provide outputs that clinicians can interpret and act upon. Prospective validation should move beyond static binary classification toward continuous, uncertainty-quantified risk scores that reflect disease stage or anticipated cognitive decline. Methodological safeguards are essential, including rigorous participant-level partitioning to prevent data leakage across multiple scans, external validation across sites and scanner vendors, and careful calibration when predicted probabilities are intended to guide clinical decisions [20]. From a clinical perspective, models that output continuous measures, such as predicted cognitive scores or atrophy-derived risk indices, are more useful than categorical labels, provided they include quantified uncertainty and clinically interpretable error margins.

PET, CSF/Plasma Biomarkers, and Genetic Data Integration

AD benefits from one of the most advanced biomarker ecosystems among neurodegenerative disorders. Amyloid and tau PET imaging provide direct biological evidence of pathology, while CSF and blood-based markers, including the amyloid-β42/40 ratio, phosphorylated tau (p-tau), neurofilament light chain (NfL), and glial fibrillary acidic protein (GFAP), capture complementary dimensions of disease biology, such as amyloid deposition, tau phosphorylation, neurodegeneration, and astroglial activation [10]. Genetic information, particularly the apolipoprotein E (APOE) genotype and polygenic risk scores, further refines risk stratification in research contexts [30].

AI methods are particularly well suited to AD because clinically relevant signals are often modest within individual modalities but become more powerful when integrated. Multimodal models that combine imaging, cognitive assessments, demographics, and biomarker panels consistently outperform single-modality approaches in tasks such as identifying prodromal AD among patients with mild cognitive impairment and predicting near-term conversion [31-33]. Importantly, the clinical value of these models lies not in headline accuracy but in their ability to reduce diagnostic ambiguity, support individualized staging, and guide timely intervention.

A major advance has been the emergence of blood-based biomarkers, particularly plasma p-tau217, which can distinguish AD from other neurodegenerative disorders in selected cohorts [34]. However, binary thresholds often yield indeterminate results in borderline cases. AI transforms these assays into dynamic diagnostic aids by contextualizing intermediate values with multimodal features, such as age, cognitive trajectories, and genetic risk. This integration generates probabilistic risk scores that support personalized triage decisions and scalable screening workflows. At the same time, careful calibration and threshold selection remain essential, as predictive value depends heavily on disease prevalence within the target population [20].

The integration of genetics and biomarkers into AI pipelines also raises governance challenges. Privacy, consent for secondary use, and equity issues, such as differences in biomarker distributions and access across populations, must be addressed to ensure fair implementation [2]. Clinical interpretability is equally critical: clinicians must be able to understand whether a risk estimate is driven by imaging, cognition, biomarkers, or demographics, and whether those contributions change in plausible directions over time. Without transparency, even technically strong models risk limited clinical adoption.

Prognostic Modeling in Alzheimer’s Disease

Prognostic modeling represents one of the most clinically meaningful applications of AI in AD, with direct implications for counseling, care planning, clinical trial enrichment, and the timely initiation of disease-modifying therapies where available [8]. Common prediction targets include conversion from MCI to dementia, trajectories of cognitive decline, and time-to-event outcomes such as institutionalization, functional milestones, or mortality [20].

Systematic reviews highlight substantial methodological variability across machine learning studies, many of which rely heavily on ADNI data and demonstrate limited external validation [32]. Multimodal models that integrate imaging, cognitive, and clinical features consistently outperform single-modality approaches, yet mean accuracies remain modest. This reflects the heterogeneity of MCI and the influence of mixed pathologies, underscoring that high discrimination alone is insufficient. Clinically useful prognostic tools must provide individualized risk estimates with quantified uncertainty, clearly defined prediction horizons, and performance stratified by age, comorbidity burden, and baseline cognitive severity [20,22].

Multimodal grading approaches that combine MRI, PET, CSF biomarkers, and genetic data have achieved predictive accuracies in the mid-80% range for conversion within defined timeframes in ADNI cohorts, although prospective validation in diverse clinical populations remains a critical gap [33]. Beyond conventional biomarkers, multi-omics integration, including genomic, transcriptomic, and micro ribonucleic acid (microRNA) profiles, has the potential to capture biological heterogeneity [35]. However, these pipelines raise concerns regarding batch effects, missing data, and overfitting in small-to-moderate samples [14,19]. They also carry substantial financial and logistical burdens, limiting their feasibility for routine triage. In contrast, scalable blood-based assays, particularly plasma p-tau species, represent a more practical solution for broad deployment, especially when contextualized by AI models that integrate multimodal features.

Ultimately, the translational value of prognostic modeling in AD lies not in headline accuracy but in its ability to generate interpretable, individualized risk estimates that inform patient-centered decisions. Success will depend on prospective validation, careful calibration, and integration into workflows that balance precision with scalability.

Risk Stratification and Clinical Applicability

Risk stratification in AD is clinically valuable because it guides the selection of individuals for confirmatory biomarker testing, identifies candidates for prevention trials, and informs the intensity of follow-up and support services [8]. However, AI-enabled risk stratification should not be viewed as a stand-alone label. Its utility is maximized when integrated within structured clinical pathways. A pragmatic model is sequential testing: low-cost screening with cognitive assessments or blood biomarkers, followed by confirmatory PET or CSF testing for individuals exceeding a defined risk threshold. Such pathways must also incorporate patient preferences and resource constraints to ensure feasibility and equity [2,34].

The ethical dimension is particularly salient in AD. Predictions carry psychological, social, and even insurance or employment implications. False positives may expose patients to unnecessary invasive testing and heightened anxiety, while false negatives risk delaying intervention [2]. For this reason, evaluation of risk stratification models must extend beyond diagnostic accuracy to include downstream effects, such as changes in clinician behavior, time to diagnosis, equity of access, and patient-centered outcomes [13,18] (Table 3).

**Table 3:** Common AD data modalities used in AI systems and typical clinical tasks AD: Alzheimer’s disease; MRI: magnetic resonance imaging; CNN: convolutional neural network; ROI: region of interest; PET: positron emission tomography; SUVR: standardized uptake value ratio; CSF: cerebrospinal fluid; Aβ: amyloid beta; p-tau: phosphorylated tau; NfL: neurofilament light chain; GFAP: glial fibrillary acidic protein; APOE: apolipoprotein E; PPV: positive predictive value

Modality	AI inputs	Representative clinical tasks and considerations
Structural MRI	Voxel/ROI features; learned 3D CNN embeddings	Diagnosis support and staging; requires external validation and leak-free splits (multiple scans per participant) [9,11,16,17,20]
Amyloid/tau PET	Uptake patterns; regional SUVRs	Biological confirmation and staging; limited by cost/access and protocol heterogeneity [10,16,20]
CSF biomarkers	Aβ42/40, p-tau, total tau	Diagnostic confidence and staging; preanalytical variability and patient acceptability influence uptake [5,10]
Plasma biomarkers	p-tau (e.g., p-tau217), NfL, GFAP	Scalable screening/triage; thresholds and PPV vary with prevalence and population characteristics [20,34]
Genetics/omics	APOE, polygenic risk, multi-omics panels	Risk stratification and prognosis; privacy, population transferability, and batch effects are key concerns [30,35]
Clinical/cognitive data	Neuropsychology batteries, demographics, comorbidities	Progression prediction and care planning; confounding and missingness require careful handling [8,20,22,32,33]

The strongest evidence for AI in AD comes from models developed using well-characterized cohorts with standardized biomarker collection, validated on independent datasets, and evaluated using clinically interpretable metrics beyond simple accuracy. At present, the key translational challenge lies not in achieving marginal improvements in area under the curve (AUC), but in demonstrating that these systems can be deployed safely, equitably, and in ways that deliver genuine clinical value in real-world memory clinics and primary care settings.

Comparative analysis of AI applications in PD and AD

Although PD and AD differ in their primary clinical features, like motor versus cognitive, and in the typical availability of disease-defining biomarkers, the AI/ML challenges in both conditions converge on three themes: the heterogeneity of phenotypes and disease trajectories, the limitations of imperfect reference standards, and the restricted generalizability of models trained on curated datasets when they are applied to real-world clinical care [14,19,20].

Data Modalities and Signal Characteristics

AI research in PD has placed strong emphasis on digital biomarkers, such as gait, tremor, handwriting, and speech, because motor features are readily observable outside the clinic and can be quantified at high frequency using wearables or smartphones [12,25,26]. By contrast, AI systems for AD more often focus on neuroimaging and fluid biomarkers, including MRI, amyloid/tau PET, and CSF or plasma assays. This is because the biological definition of AD is increasingly anchored in pathology-based frameworks, and subtle cognitive changes are difficult to capture passively without the use of standardized testing [10,34]. Nevertheless, both fields are moving toward multimodal fusion, integrating imaging and biomarker data with clinical measures to enhance robustness and reliability [28,33].

Model Families and Evaluation Targets

In both PD and AD, traditional ML models are commonly applied when inputs are structured (e.g., diffusion indices, SUVRs, and kinematic features). Deep learning, by contrast, is the dominant approach for end-to-end analysis of raw or minimally processed data, such as three-dimensional (3D) MRI volumes, video, or audio [11]. The clinical targets, however, differ between the two conditions. PD studies typically emphasize early diagnosis, distinguishing PD from other parkinsonian syndromes, including atypical syndromes, quantifying symptoms, and detecting medication-related fluctuations [15,24]. AD studies more often focus on classification (AD vs. controls; AD vs. MCI), disease staging, and prediction of conversion from MCI to AD. This reflects AD’s long prodromal phase and the clinical importance of forecasting decline to guide care planning [32,33].

Reference Standards and Ground Truth

Both PD and AD research face challenges with label noise when clinical diagnoses are used as reference standards. In PD, misdiagnosis is common during early stages and in atypical parkinsonism, while changes in diagnosis over follow-up further complicate model training and evaluation [7]. In AD, labels based on clinical syndromes may not align with biomarker-confirmed disease biology, and mixed pathologies are frequent among older adults. As a result, model performance can vary significantly depending on whether labels are derived from clinical assessments or supported by biological biomarkers [8,10].

Generalizability, Bias, and Calibration

Dataset shift is a major limitation for AI systems in both PD and AD. In imaging, scanner and vendor differences, along with site-specific preprocessing, can produce overly optimistic internal validation results. For digital biomarkers, variability in devices, user adherence, and contextual factors, such as footwear, environment, language, or microphone quality, can degrade performance once these systems are deployed [2,12]. Consequently, external validation and transparent reporting are critical but remain inconsistently applied across studies [13,19]. Calibration is especially important in risk prediction tasks, such as identifying high-risk MCI patients or triaging suspected parkinsonism, because poorly calibrated probabilities may trigger unnecessary testing or cause undue anxiety [20].

Clinical Implementation and Ethical Considerations

AI systems for PD may be introduced earlier in the care pathway because sensor-based tools are relatively low-cost and scalable. However, unequal access to smartphones or wearables could create inequities. In AD, AI approaches are likely to increasingly rely on blood-based biomarkers combined with machine learning for triage. Yet, the predictive use of AI in AD carries greater psychosocial risks, making careful consent, governance, and threshold setting essential. Across both conditions, the near-term value of AI is best understood as an augmentation strategy, providing standardized measurement, decision support, and longitudinal monitoring, rather than replacing clinical assessment (Table 4).

**Table 4:** Comparison of AI models used in Parkinson’s and Alzheimer’s disease PD: Parkinson’s disease; AD: Alzheimer’s disease; MRI: magnetic resonance imaging; DaT-SPECT: dopamine transporter single photon emission computed tomography; PET: positron emission tomography; FDG-PET: fluorodeoxyglucose positron emission tomography; LRRK2: leucine-rich repeat kinase 2; SNCA: alpha-synuclein gene; GBA: glucocerebrosidase gene; APOE: apolipoprotein E; CNN: convolutional neural network; ROI: region of interest; PPV: positive predictive value

Category	Parkinson’s disease (PD)	Alzheimer’s disease (AD)
Imaging	MRI (structural/functional), DaT-SPECT for dopaminergic deficit, gait-related imaging [7,15,18]	Structural MRI, amyloid/tau PET, FDG-PET for metabolism, multimodal imaging integration [9,10,16,20]
Digital biomarkers	Wearable sensors (accelerometry, gyroscopes), gait analysis, voice/speech recordings, typing/motor tasks [12,22,24, 25]	Smartphone-based cognitive testing, digital speech/language analysis, passive monitoring via wearables [23,32]
Genetic biomarkers	LRRK2, SNCA, GBA variants; polygenic risk scores [30]	APOE ε4, polygenic risk scores, multi-omics (genomics, proteomics, metabolomics) [30,35]
Outcomes	Motor symptom quantification, tremor/bradykinesia scoring, fall risk prediction, medication response monitoring [12,14,22]	Cognitive decline detection, staging, progression prediction, risk stratification, trial enrichment [20,32,33]
Key challenges	Variability in motor symptoms, medication effects, need for longitudinal datasets [2,14,19]	Confounding comorbidities, biomarker variability, assay harmonization, population heterogeneity [8,10,34]
Clinical utility	Remote monitoring, personalized therapy adjustments, early detection of motor complications [12,24,25]	Early diagnosis, scalable screening, care planning, clinical trial enrichment [20,34]
Validation considerations	Device standardization, multicenter datasets, external validation [14,17]	Leak-free splits, biomarker assay harmonization, population transferability [20,32,33]

Clinical implications of AI models

Earlier Diagnosis and Differential Diagnosis

The most immediate clinical opportunity for AI in neurodegenerative disorders lies in improving the timeliness and accuracy of diagnosis, particularly in settings with limited access to specialists or advanced biomarker testing. Imaging-based models that aid differential diagnosis, such as those distinguishing between parkinsonian syndromes, may help reduce delays and connect patients earlier with appropriate prognostic counseling and supportive therapies [7,15]. In AD, AI systems that combine MRI with biomarker data can strengthen diagnostic confidence in borderline cases and guide patient selection for confirmatory testing when resources are limited [10,34]. Crucially, the aim of these pathways should be to reduce diagnostic uncertainty and avoid unnecessary testing, rather than replace clinical judgment. Even modest gains in triage accuracy, paired with clearer referral thresholds, could deliver meaningful downstream benefits by shortening time-to-diagnosis.

Personalized Medicine and Prognostic Enrichment

Predictive modeling can enhance individualized care planning by estimating near-term progression risk, expected symptom trajectories, and the likelihood of clinically meaningful milestones [20,33]. In practice, these estimates can guide follow-up intensity, trigger early referrals for rehabilitation or caregiver support, and improve clinical trial enrollment by identifying patients most likely to progress within the study period [8]. For such predictions to be clinically useful, models must be calibrated and validated in the populations where they will be applied, such as primary care or specialty clinics, because predictive values depend heavily on prevalence and case mix [20]. In AD especially, where blood biomarkers and AI-driven risk estimates may be deployed at scale, implementation should emphasize shared decision-making and transparent communication of uncertainty to minimize potential psychological harms [2].

Remote Monitoring and Outcome Measurement

Digital biomarkers enable more frequent and objective tracking of motor and functional status. In PD, wearables and smartphones can capture gait, tremor, and speech, enhance the detection of medication-related fluctuations, and provide more sensitive endpoints for interventional studies [12,24]. In AD, passive monitoring and repeated cognitive tasks may eventually complement biomarker-based staging, although the evidence base is less developed and privacy concerns are greater [2]. 

Operational Realities of AI Models

The real-world utility of AI architectures in neurodegenerative disease management depends on their ability to move beyond static algorithms and be integrated into scalable engineering and clinical workflows [2]. These systems are not designed to function as isolated diagnostic tools but rather as automated assistive layers that standardize high-frequency measurements and streamline patient triage pathways [13,21]. By embedding themselves into routine care, they enhance efficiency and consistency in the management of complex conditions.

In PD, ML frameworks are applied to continuous, high-frequency data streams collected from wearable devices [36,37]. Wrist-worn inertial measurement units and smartphones capture accelerometry and gyroscopic time-series data during daily activities, providing a passive yet rich source of information [12,21-23]. Ensemble pipelines, such as Particle Swarm Optimization-Random Forest models, then extract detailed motor parameters, gait changes, and tremor variability outside traditional clinical settings [36-39]. Longitudinal telemetry systems, validated by multicenter studies such as Wearable Assessments in the Clinic and Home in Parkinson's Disease (WATCH-PD), transform these raw behavioral signals into objective metrics that track treatment responsiveness and early motor complications in real-world environments [40].

For AD, operational frameworks emphasize cloud-based computational fusion to address the challenge of long prodromal phases that require early intervention [41,42]. These decentralized platforms automate screening through tiered triage pathways. Deep learning models integrate multimodal data by combining home-based cognitive tasks, eye-tracking, and finger-stick blood assays with neuroimaging variables [41-44]. By contextualizing plasma profiles with behavioral and clinical time-series features, the computational layer converts static biomarkers into dynamic predictors of amyloid and tau PET profiles [43,44]. Automated screening platforms, such as those implemented in community-based initiatives like PREDICTOM, filter decentralized data streams to identify patients whose physiological trajectories deviate significantly from baseline [45]. This approach ensures that invasive procedures and costly neuroimaging are reserved for individuals with a higher probability of disease, thereby optimizing specialist resource allocation and improving the efficiency of early detection [45].

Current limitations and future challenges of clinical implementation

Bias, Fairness, and Transportability Barriers

A pervasive limitation hindering the translation of machine learning pipelines into clinically reliable SaMD is the systemic overreliance on highly curated, demographically uniform datasets [14,17,19,38,39,46]. This homogeneity inflates performance metrics during internal testing but severely undermines real-world generalizability [14,17,19]. Even landmark multi-center initiatives, such as the WATCH-PD study, frequently lack the clinical and demographic diversity required for broad geographical transportability [40]. When deployed within heterogeneous community clinics, these algorithms encounter significant transportability barriers as discussed below.

Baseline confounding: Age-related physical and neurological co-morbidities, such as arthritis, peripheral neuropathy, vascular disease, clinical depression, and concurrent metabolic or structural conditions, frequently distort speech, handwriting, and gait metrics [2,8,10,14,24,25,34]. These overlapping clinical features introduce systemic noise, often triggering high rates of false positives [2,14].

Missing data matrices: Real-world clinical datasets are rarely complete, and high-dimensional genomics, proteomics, metabolomics, and other (OMICS) pipelines suffer from substantial missing data structures, batch effects, and small-to-moderate sample sizes that can induce severe overfitting [14,19,20,32,33,35].

Device and protocol heterogeneity: For digital biomarkers, variations in user adherence, device placement, hardware specifications, and environmental conditions (e.g., microphone quality, footwear) degrade algorithmic performance outside highly controlled settings [2,12,14].

Interpretability, Label Noise, and Medico-Legal Realities

A foundational challenge to robust validation in early-stage neurodegenerative disease is the absence of a definitive biological ground truth [3,6,7,27]. Evolving clinical diagnoses, coupled with high inter-rater variability in subjective clinical scoring scales, such as the Movement Disorder Society-Sponsored Revision of the Unified Parkinson's Disease Rating Scale (MDS-UPDRS), introduce substantial label noise into training pipelines [3,6,7,14,27]. When DL networks are trained on ambiguous or misclassified prodromal labels, their mathematical accuracy is fundamentally capped, creating a misleading performance ceiling that fails to reflect underlying pathobiology [7,8,14].

Furthermore, end-to-end DL architectures applied to raw audio spectrograms or three-dimensional neuroimaging volumes function as opaque "black boxes" [9,11,13]. Clinicians cannot safely act on uninterpretable binary outputs; safe translation demands explicit uncertainty quantification, probabilistic risk distributions, and region-level feature attributions [13,18,20]. Without these interpretability safeguards, models fail to gain clinician trust and run afoul of medical-legal accountability standards [13].

Workflow Integration and Infrastructure Instability

The operational reality of multi-modal AI frameworks in neurology is highly vulnerable to localized hardware and infrastructure shifts [2,14,47]. For example, routine clinical modifications, such as upgrading structural imaging infrastructure from a standard 1.5T to a 3T MRI magnet, alter tissue contrast, signal-to-noise ratios, and artifact patterns [14]. These shifts instantly degrade the diagnostic accuracy of deep learning models calibrated on older, low-resolution data [14].

Finally, translation remains bottlenecked by a lack of prospective evidence [2,4,8,13]. Current validation strategies rely heavily on retrospective single-metric discrimination, like the AUC, rather than prospective, multi-site pragmatic trials that evaluate net clinical benefit [13,17,20,34,44,48]. Until AI layers are framed as assistive augmentation tools within tiered triage workflows and evaluated using standardized reporting frameworks such as CONSORT-AI and TRIPOD-AI on downstream outcomes like time-to-diagnosis and patient-centered metrics, real-world clinical adoption will remain severely restricted [13,17,21,48].

Data Infrastructure and Governance

Scalable clinical AI in neurology depends on robust data infrastructure: standardized acquisition protocols (e.g., imaging sequences, biomarker assays, device placements), versioned preprocessing pipelines, and consistent outcome definitions [14,19]. Federated or multi-institutional collaborations can reduce overfitting to single cohorts and improve representativeness, but they also require strong governance, transparent consent processes, and strict privacy protections, particularly for sensitive modalities such as voice recordings and passive behavioral data [2]. Preserving data provenance (where and how data were collected) is essential to enable auditing and to support root-cause analysis when model performance declines.

For AI to move beyond proof-of-concept studies and achieve meaningful clinical impact in neurodegenerative diseases, certain practical requirements must be met. These requirements encompass not only technical aspects such as dataset size, longitudinal monitoring, and standardization, but also clinical integration, external validation, and adherence to ethical and regulatory frameworks. Addressing these factors is critical to ensure that AI systems for PD and AD are reliable, generalizable, and acceptable to both clinicians and patients. The following table outlines the key prerequisites that must be satisfied for deployment-ready AI in these conditions. Despite the rapid progress of AI in supporting the diagnosis and management of neurodegenerative diseases, several limitations remain that hinder its widespread clinical adoption.

These challenges span technical, clinical, and ethical domains, including data heterogeneity, small sample sizes, confounding comorbidities, and variability in biomarker assays. Moreover, issues of generalizability, patient adherence, cost barriers, and regulatory concerns continue to restrict scalability and real-world integration. Understanding these limitations is essential to contextualize current findings and to guide future research toward more robust, equitable, and clinically meaningful AI applications. Addressing these multi-domain hurdles is critical to ensure that AI systems for PD and AD are reliable, generalizable, and clinically acceptable. The following consolidated framework outlines these practical requirements, technical limitations, and translation challenges side-by-side (Table 5).

**Table 5:** Practical requirements and key challenges for deployment-ready AI in PD and AD PD: Parkinson’s disease; AD: Alzheimer’s disease; AI: artificial intelligence; PET: positron emission tomography; CSF: cerebrospinal fluid; OMICS: collective term for genomics, proteomics, metabolomics, etc.; ENT: ear, nose, and throat

Category	Parkinson’s disease (PD)	Alzheimer’s disease (AD)
Core infrastructure and data scope		
Large, diverse datasets	Multicenter cohorts with standardized motor assessments and imaging protocols [14,17,19]; limited by small, single-center datasets that hinder broad generalizability [14,17,19]	Population-diverse cohorts with harmonized biomarker assays and cognitive batteries [20,32,33,35]; limited by low data transferability across populations [20,32,33,35]
Longitudinal data and tracking	Continuous tracking of motor fluctuations, medication response, and disease progression outside episodic clinic visits [12,22]	Long-term follow-up for cognitive decline trajectories, clinical milestone forecasting, and structural/fluid biomarker staging [8,10,34]
Data and phenotypic heterogeneity	Addressing clinical variability in motor symptom presentation, medication effects, device placement variations, and patient adherence [12,14,22]	Navigating structural variability across multi-site biomarker assays, imaging protocols, and cognitive test batteries [8,10,20,34]
Cohort scale and overfitting	Vulnerable to inflated performance metrics due to highly curated, single-center datasets lacking clinical diversity [14,17,19]	Constraints involving small-to-moderate multi-omics samples, missing data matrices, and a lack of geographically distinct validation cohorts [14,19,20,32,33,35]
Methodological and technical safeguards		
Standardization and calibration	Cross-scanner normalization, sensor calibration across varied hardware, and strict wearable positioning frameworks [15,18]	Standardization of fluid assays, harmonized imaging protocols, and batch correction frameworks for high-dimensional omics data [9,16,30,35]
External validation	Testing models on independent datasets using leak-free splits to ensure reproducibility across distinct clinics [14,17]	Rigorous validation across diverse demographic settings and independent biomarker datasets to ensure transportability [20,32,33]
Confounding factors	Distinguishing target features from age-related confounders, arthritis, peripheral neuropathy, and concurrent ENT conditions [24,25]	Factoring out concurrent vascular disease, clinical depression, and age-related metabolic or structural comorbidities [8,10,34]
Workflow, ethics, and clinical translation		
Electronic health record and workflow integration	Clinician-friendly telemetry interfaces and explainable metrics integrated directly into electronic health records to avert alert fatigue [19,22]	Clinical decision support platforms aligned with trial enrichment pipelines, sequential triage workflows, and long-term care planning [20,34]
Clinical adoption and access barriers	Patient adherence hurdles with remote hardware, combined with high costs and restricted access to confirmatory nuclear imaging [12,18]	Substantial financial barriers for amyloid/tau PET, assay accessibility gaps, and patient hesitancy regarding invasive lumbar punctures [5,10,20]
Ethical and regulatory compliance	Safeguarding continuous passive behavioral data streams, establishing robust informed consent, and securing SaMD clearance [12,18,19,22]	Preserving strict data privacy for genetic/OMICS profiles, resolving "black box" deep learning opacities, and navigating regulatory SaMD approval [30,35]

Study limitations

This review offers a comprehensive and structured synthesis of AI applications in neurodegenerative disease management, but certain boundary conditions of its scope must be acknowledged. First, the work is organized as a narrative review rather than a formal systematic review and/or meta-analysis. Given the heterogeneity of data modalities, preprocessing methods, and algorithmic architectures across the evaluated literature, a direct quantitative pooling of performance metrics, such as a unified meta-regression of sensitivity or specificity, was neither feasible nor methodologically appropriate. Second, the findings presented are inevitably shaped by the current state of published literature, which tends to emphasize optimized performance metrics while underreporting null or replication outcomes. Third, much of the foundational data in this field is derived from standardized open-access repositories such as ADNI or collaborative cohorts like the WATCH-PD study. While these datasets have been instrumental in driving early algorithmic innovation, they represent highly structured environments, and the generalizability of these models to diverse, unaligned global clinical populations remains an active area of investigation. Finally, because standardized clinical reporting frameworks such as CONSORT-AI and TRIPOD-AI are relatively recent consensus developments, earlier landmark studies often vary in their reporting structures. This necessitates a contextualized narrative interpretation of validation methodologies in older works, ensuring that their contributions are understood within the evolving landscape of reporting standards.

## Conclusions

AI and ML are redefining how neurodegenerative disorders are studied and managed, but their clinical translation depends on tailoring approaches to the distinct biological and structural demands of each disease. In AD, the challenge lies in its prolonged prodromal phase and complex biological heterogeneity. Here, AI adds value by fusing structural imaging, fluid biomarkers, and genetic or omics data into longitudinal risk-stratification pipelines. These integrative models help forecast progression from mild cognitive impairment to dementia, refine patient selection for clinical trials, and guide early intervention pathways for emerging disease-modifying therapies. In PD, translational progress is best aligned with continuous, high-frequency behavioral monitoring. Motor symptoms are visible, fluctuate with treatment cycles, and lend themselves to digital phenotyping through wearables, smartphones, and automated analyses of speech or handwriting. When combined with ML-based neuroimaging support, these tools provide scalable augmentation for early screening, symptom tracking, and differentiation of idiopathic PD from atypical parkinsonian syndromes in community care.

Despite promising advances, high accuracy scores from curated research datasets do not equate to clinical readiness. Both PD and AD applications remain vulnerable to dataset shifts, device variability, and evolving patient profiles. To safeguard patients and avoid harms from misclassification, AI should be framed as an assistive augmentation layer rather than a diagnostic replacement. Scalable adoption will require prospective multicenter validation, transparent reporting of model uncertainty, and robust governance frameworks that prioritize equity, privacy, and accountability.

## Appendices

Comprehensive compliance mapping tables demonstrating the integration of these dual frameworks within our systematic synthesis are provided in Table 6 and Table 7.

**Table 6:** CONSORT-AI checklist CONSORT-AI: Consolidated Standards of Reporting Trials-Artificial Intelligence

CONSORT-AI item	Extension description	Manuscript alignment/status
Item 1a: Title/Abstract	Identification in the title or abstract that an AI intervention is being evaluated	Fully Aligned. Abstract explicitly references machine learning, deep learning, and multimodal frameworks for clinical workflows
Item 5i: Intervention	Clear description of the AI software, hardware, versioning, and operational requirements	Fully Aligned. Detailed across Section "Software as a Medical Device" and Table 4 (Prerequisites)
Item 5ii: Data Input	Specification of the minimum data quality and acquisition parameters required to run the AI	Fully Aligned. Outlined for MRI protocols, fluid assays, and sensor data in Tables 2 and 3
Item 5iii: Human-AI Interaction	Description of how the AI outputs are presented to the clinician and integrated into the workflow	Fully Aligned. Section "Workflow Integration" explicitly defines the tool as an assistive augmentation layer
Item 19i: Out-of-Specification	Documentation of known failure modes, data exclusions, and out-of-specification inputs	Fully Aligned. Detailed in Section "Interpretability and Legal Considerations" (e.g., severe comorbidities, noise)

**Table 7:** TRIPOD-AI checklist TRIPOD-AI: Transparent Reporting of Multivariable Prediction Model for Individual Prognosis or Diagnosis-Artificial Intelligence

TRIPOD-AI item	Standard reporting requirement	Manuscript alignment/status
Item 1: Title and Abstract	Identify the study as developing or validating a prediction model using AI/ML	Fully Aligned. Explicitly specified throughout the review methodology and progression targets
Item 4: Source of Data	Describe the data sources used to develop and test the model (e.g., ADNI, multi-center cohorts)	Fully Aligned. Data sources used to develop and test the models (including ADNI, the WATCH-PD cohort, and the PREDICTOM framework) are critically evaluated across the disease-specific imaging and fluid biomarker subsections. Their methodological generalizability and multi-center validation constraints are further synthesized within the section titled 'Current limitations and future challenges of clinical implementation'
Item 10: Model Development	Specify data partitioning strategies and participant-level handling to avoid data leakage	Fully Aligned. Addressed as a core critical appraisal metric in "Comparative Analysis" and Table 4
Item 11: Performance Metrics	Report both discrimination (AUC, sensitivity) and calibration (uncertainty, probability fit)	Fully Aligned. Sections "Generalizability & Calibration" emphasize moving past simple summary scores
Item 15: External Validation	Evaluate the model's performance on independent, geographically distinct datasets	Fully Aligned. Set as a primary prerequisite across all discussed modalities in Section "Future Directions"
